# Outpatient Care Among Users and Nonusers of Direct-to-Patient Telehealth: Observational Study

**DOI:** 10.2196/37574

**Published:** 2022-06-06

**Authors:** Alison Cuellar, J Mary Louise Pomeroy, Sriteja Burla, Anupam B Jena

**Affiliations:** 1 Department of Health Administration and Policy George Mason University Fairfax, VA United States; 2 National Bureau of Economic Research Cambridge, MA United States; 3 Department of Economics George Mason University Fairfax, VA United States; 4 Department of Health Care Policy Harvard Medical School Boston, MA United States; 5 Department of Medicine Massachusetts General Hospital Boston, MA United States

**Keywords:** telemedicine, insurance, policy, telehealth, user, primary care, outpatient, claims, in-person, virtual, insurer, coverage

## Abstract

**Background:**

Expansion of telehealth insurance coverage is hampered by concerns that such coverage may encourage excessive use and spending.

**Objective:**

The aim of this paper is to examine whether users of telehealth services rely more on other forms of outpatient care than nonusers, and to estimate the differences in payment rates.

**Methods:**

We examined claims data from a large national insurer in 2017. We limited our analysis to patients with visits for 3 common diagnoses (N=660,546). We calculated the total number of visits per patient, overall, and by setting, and adjusted for patient- and county-level factors.

**Results:**

After multivariable adjustment, telehealth-visit users, compared to nonusers, had 0.44 fewer visits to primary care, 0.11 fewer visits to emergency departments, and 0.17 fewer visits to retail and urgent care. All estimates are statistically significant at *P*<.001. Average payment rates for telehealth visits were lower than all other settings.

**Conclusions:**

These findings suggest that telehealth visits may substitute rather than add to in-person care for some types of care. Our study suggests that telehealth visits may offer an efficient and less costly alternative.

## Introduction

Telemedicine has dramatically changed health care delivery since COVID-19, offering safety and convenience [1]. To promote greater access to care, federal and state policy makers temporarily removed several telehealth barriers, including those related to insurance coverage and reimbursement [2,3]. However, concerns exist about making these changes permanent. On the one hand, telehealth has the potential to improve access to care (eg, by helping patients to overcome barriers such as transportation and childcare). On the other hand, expansion of telehealth insurance coverage is hampered by concerns that such coverage may encourage excess use and spending. Such concerns are heightened when payment is on a per-visit, fee-for-service basis [4].

Most direct-to-patient (DTP) telehealth visits have been found to occur outside of regular business hours, suggesting that convenience and accommodation may be key considerations [5]. In a survey of adults conducted in 2019 before the COVID-19 pandemic, 49% of adults reported being willing or very willing to use video visits [6]. Previous studies have found that DTP telehealth users are more likely to live in urban areas, be younger, and less likely to have comorbid conditions than the general population, indicating that access and affordability may not be the key drivers of telehealth visit use [5]. Evidence is limited with respect to the potential for telehealth visits to serve as a substitute for in-person care rather than as a complement to it. If telehealth visits substitute for in-person care, this could mean health care savings; however, if telehealth visits complement and add to in-person visits, then this would increase health care expenditures. Early evidence for telehealth acute respiratory illnesses found that telehealth visits represented additional use rather than replacing visits to other providers [7]. A more recent study of telehealth found no differences in total outpatient visits after hospital discharge, early in the pandemic [8]. In a survey of people experiencing homelessness, 29.1% self-reported they would have sought care in an emergency department (ED) if they had not had access to telehealth [9]. Additional evidence on whether telehealth complements or substitutes for in-person care is lacking.

We examined claims data from a large national insurer that offered DTP telehealth visits as a covered benefit. We assessed whether telehealth visits were associated with differences in the use of office-based primary care, retail and urgent care clinics, and EDs.

## Methods

### Data and Measures

Our cross-sectional study used 2017 private insurance claims data for continuously enrolled members ages 18-64 years who were offered telehealth services through a third-party DTP vendor. We limited our analysis to the top 3 most common claims diagnoses for telehealth visits in order to increase comparability across patients and sites. The most common diagnoses were respiratory infections, diseases of the urinary system, and eye disorders. We further limited our analysis to telehealth visits, office-based primary care, urgent or retail clinics, and EDs based on claim codes. We calculated the total number of visits per member who experienced at least one visit with the target diagnoses. Total visits were calculated overall and by setting. Separately, we calculated mean insurer paid amounts for evaluation and management visits for each setting.

### Analysis

We estimated patient-level multivariable, negative binomial regression models in which the dependent variable was the total number of visits in a given care setting (separate regressions were run for primary care, urgent or retail clinics, and ED visits). Independent variables included an indicator for having had a telehealth visit that year, an indicator for having high-deductible health insurance coverage, and an indicator for having had a primary care office visit in the prior year. Additional control variables included age, sex, a continuous measure of illness burden using claims-based Episode Treatment Groups, a rural county indicator, county-level measures of total primary care physicians and EDs obtained from claims, and county-level counts of retail clinics and urgent care centers. We also adjusted for county-level demographics and commute times from the American Community Survey. For each visit type, we calculated regression-adjusted total visits by whether the member had a telehealth visit. Analysis was conducted using Stata (Version 15, StataCorp).

### Ethics Approval

The National Bureau of Economic Research institutional review board determined this study to fall under Exemption #4 as detailed at 45 CFR Part 46 Subpart A Section 46.101 [10]. As such, it has been exempted from review.

## Results

Overall, 660,546 members with the selected diagnoses had, on average, 0.56 visits for primary care, 0.60 for retail or urgent care centers, 0.13 for EDs, and 0.04 for telehealth care in 2017 (Table 1). The median insured paid amounts were US $40 for telehealth visits, US $87 for primary care, US $113 for urgent and retail clinics, and US $812 for ED visits.

**Figure 1:**
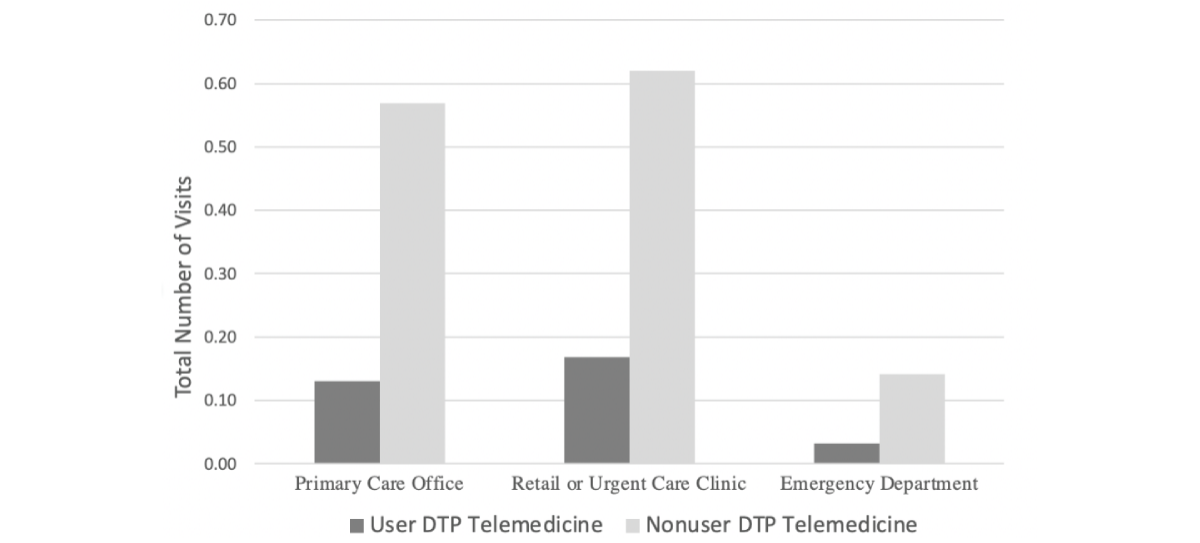
Outpatient use for direct-to-patient (DTP) telemedicine users and nonusers.

After multivariable adjustment (Figure 1), telehealth-visit users had fewer visits to primary care compared with nonusers (0.13 vs 0.57, adjusted difference 0.44; 95% CI 0.44 to 0.45), EDs (0.03 vs 0.14, adjusted difference 0.11; 95% CI 0.11 to 0.11), and retail and urgent care (0.17 vs 0.62, adjusted difference 0.45; 95% CI 0.45 to 0.46). These estimates of number of visits control for the individual having a primary care visit in the prior year, age, gender, illness burden measured from claims-based Symmetry groupings, whether their insurance plan had a high deductible, rural status, and other county characteristics of the patient. The sample includes patients with respiratory infections, diseases of the urinary system, and eye disorders. All estimates are statistically significant at *P*<.001.

**Table 1:** Characteristics of the study population.

Annual visits and population characteristics		All (N=660,546)	Telehealth visit nonuser (N=639,975)	Telehealth visit user (N=20,571)	Difference (95% CI)
**Annual visits**					
	Primary care visits	0.56	0.57	0.11	0.46 (0.45, 0.47)
	Retail and urgent visits	0.60	0.62	0.18	0.44 (0.43, 0.45)
	ED^a^ visits	0.13	0.13	0.03	0.10 (0.10, 0.11)
**Individual-level characteristics**					
	Age (years)	41.5	41.6	39.3	2.3 (2.2, 2.5)
	Female, n (%)	410,199 (62.1)	396,785 (62.0)	13,515 (65.7)	–3.7 (–4.3, –3.0)
	Primary care office visit, prior year, n (%)	280,732 (42.5)	274,549 (42.9)	6151 (29.9)	13.1 (12.5, 13.7)
	High deductible health plan, n (%)	292,622 (44.3)	280,949 (43.9)	11,911 (57.9)	–14.0 (–14.7, –13.3)
	Illness burden score	1.76	1.77	1.51	0.25 (0.23, 0.28)
**County-level characteristics**					
	White, n (%)	480,877 (72.8)	465,902 (72.8)	15,140 (73.6)	–0.8 (–1.0, –0.6)
	<18 years of age, n (%)	153,907 (23.3)	149,114 (23.3)	4875 (23.7)	–0.4 (–0.4, –0.3)
	>65 years of age, n (%)	93,798 (14.2)	90,876 (14.2)	2859 (13.9)	0.3 (0.3, 0.4)
	Bachelor’s degree or higher, n (%)	227,228 (34.4)	220,791 (34.5)	6974 (33.9)	0.5 (0.4, 0.7)
	Median household income (in thousands of US $)	65.2	65.3	64.1	1.2 (0.9, 1.4)
	Private insurance, n (%)	457,098 (69.2)	442,863 (69.2)	14,132 (68.7)	0.5 (0.4, 0.7)
	Rural county, n (%)	7927 (1.2)	7680 (1.2)	226 (1.1)	0.2 (0.0, 0.3)
	Mean travel time to work (min)	27.3	27.3	26.8	0.45 (0.39, 0.52)
	Primary care providers per 1000 members	8.63	8.66	7.79	0.86 (0.1, 0.1)
	Retail and urgent care per 100,000 population	4.42	4.42	4.41	0.03 (–0.03, 0.05)^b^
	EDs per 100,000 population	0.776	0.775	0.785	0.010 (–0.021, 0.001)^b^

^a^ED: emergency department.

^b^All differences had *P* values <.001 except these measures where *P*<.05.

## Discussion

Among the beneficiaries of a large insurer treated for 1 of 3 common acute outpatient conditions, telehealth visit users had lower use of primary care, retail clinic and urgent care, and ED visits for those conditions. These findings suggest that telehealth visits may substitute rather than add to in-person care in some settings, although the extent of substitution or addition is unknown due to the possibility of unmeasured confounders.

In this study, insurer payment rates for third-party, DTP telehealth visits were lower than payment rates for visits in other settings, although the scope of care was narrow. Prior to the pandemic, 6 states had telemedicine parity laws, which mandated that private insurers reimburse telehealth visits on par with in-person visits [11]. By fall 2021, a total of 21 states had reimbursement parity laws for commercial insurance [12]. As these parity laws have become more common, they reduce the payment differences between in-person care and care delivered by DTP telemedicine networks, lowering potential savings to insurers.

Use of all forms of telemedicine is increasingly rapidly, offering the potential for significant improvements in health care access [13], particularly as greater investments are made to expand broadband access for rural areas and low-income families through recently passed federal legislation. Our study suggests that for some conditions, telehealth visits may be an efficient and less costly alternative to care in other settings.

Study limitations include analysis of a large, national insurer in a single year, a specific telehealth service, and select conditions, which may not generalize or apply to more specialized forms of telemedicine, including recent telehealth care that has been audio only. Commercial claims data also do not contain racial and ethnic information. Further, our study was observational and measured associations between telehealth visits and other forms of visit. Future work should examine other forms of televisit and additional patient populations.

## Abbreviations

DTP: direct-to-patient
ED: emergency department
